# Antioxidant Supplement Inhibits Skeletal Muscle Constitutive Autophagy rather than Fasting-Induced Autophagy in Mice

**DOI:** 10.1155/2014/315896

**Published:** 2014-06-15

**Authors:** Zhengtang Qi, Qiang He, Liu Ji, Shuzhe Ding

**Affiliations:** ^1^Key Laboratory of Adolescent Health Assessment and Exercise Intervention, Ministry of Education, East China Normal University, Shanghai 200241, China; ^2^College of Physical Education and Health, East China Normal University, Shanghai 200241, China

## Abstract

In this study, we tested the hypothesis that NAC administration leads to reduced oxidative stress and thus to decreased expression of autophagy markers in young mice. Our results reveal that NAC administration results in reduced muscle mRNA levels of several autophagy markers, including Beclin-1, Atg7, LC3, Atg9, and LAMP2. However, NAC supplement fails to block the activation of skeletal muscle autophagy in response to fasting, because fasting significantly increases the mRNA level of several autophagy markers and LC3 lipidation. We further examined the effects of NAC administration on mitochondrial antioxidant capacity in fed and 24-hour fasted mice. Our results clearly show that NAC administration depresses the expression of manganese superoxide dismutase (MnSOD) and TP53-induced glycolysis and apoptosis regulator (TIGAR), both of which play a predominant antioxidant role in mitochondria by reducing ROS level. In addition, we found no beneficial effect of NAC supplement on muscle mass but it can protect from muscle loss in response to fasting. Collectively, our findings indicate that ROS is required for skeletal muscle constitutive autophagy, rather than starvation-induced autophagy, and that antioxidant NAC inhibits constitutive autophagy by the regulation of mitochondrial ROS production and antioxidant capacity.

## 1. Introduction

Autophagy, a conserved intracellular process carrying out degradation of organelles and proteins, has been linked to apoptosis and cell death, serving both muscle growth and mitochondrial quality control [1, 2]. Autophagy-related genes (Atgs) are required for autophagic signaling pathway and the formation of autophagosome. Beclin-1 (Atg6) is an important component of the Class III phosphoinositide 3-kinase (PI3K) complex, which plays a positive role in the initial step of autophagosome formation. Beclin-1 recruits the other Atg proteins to the Class III PI3K complex and thus starts the next step of autophagy [3]. Atg7, Atg9, and LC3 all play important roles in elongation and formation of the autophagosome [4]. LC3-II, the lipidated form of LC3, was identified as an intrinsic autophagosomal membrane marker in autophagy [5]. Finally, lysosomal-associated membrane protein-2 (LAMP-2) is responsible for the fusion of autophagosomes with lysosomes. LAMP-2 deletion causes the accumulation of autophagosomes and a decrease in autolysosomes [6].

Autophagy can be carried out in stressed or unstressed state. Under conditions of stress, such as nutrient deprivation, starvation, and hypoxia, autophagy allows cells to survive by releasing energy substrates via degradation of cellular constituents. In the stressed state of fasting, the marker proteins for autophagy were increased in skeletal muscle. Further, the autophagic response preferentially occurred in fast-switch muscle during fasting; this difference in autophagy regulation resulted from differential signaling transduction in slow- and fast-twitch skeletal muscle [7, 8]. In unstressed state, autophagy contributes to the elimination of damaged organelles and proteins; therefore, impaired autophagy is implicated in the pathogenesis of heart disease, diabetes, obesity, neurodegenerative disease, and cancer [9]. Autophagy plays a dual role in cancer, acting both as a tumor suppressor by preventing the accumulation of damaged proteins and organelles and as a tumor promoter by promoting cell survival [10]. In skeletal muscle, autophagy is required to maintain muscle mass and mitochondrial function [11]; impaired autophagy contributes to muscle atrophy in glycogen storage disease [12]. Although autophagy is increased during muscle wasting, and the induction of autophagy precedes muscle loss [13], inhibiting autophagy is unable to protect skeletal muscle from atrophy during denervation and fasting; instead it promotes greater muscle loss [14]. These findings indicate that skeletal muscle autophagy at the basal level is both detrimental, to cause muscle protein degradation, and beneficial, to promote muscle cell survival and mitochondrial quality control. A complete understanding of the mechanisms responsible for skeletal muscle autophagy in stressed and unstressed state remains unclear.

Regardless of being in the stressed state and at the basal level, autophagy induction is involved in reactive oxygen species (ROS) and cellular redox regulation. Mitochondria play an important role in the regulation of autophagy by generating endogenous ROS on one hand and scavenging ROS on the other hand [15]. Thus, maintaining mitochondrial homeostasis by eliminating defective mitochondria is required for the maintenance of redox homeostasis [16]. Under oxidative stress, defective autophagy may also induce abnormal mitochondrial accumulation. In contrast to the increase in mitochondrial mass, mitochondrial DNA was observed to decrease with mitochondrial dysfunction under the oxidative conditions in cardiac myoblast cells [17]. Compared with fragmented mitochondria, elongated mitochondria are more resistant to ROS-induced damage and mitochondrial autophagy (mitophagy); ROS-induced mitochondrial damage may be an important upstream activator of mitophagy [18]. In many cell types, cucurbitacin-induced autophagy was mediated by increased production of mitochondrial-derived ROS and subsequently activation of extracellular signal-regulated kinase (ERK) and c-jun NH2-terminal kinase (JNK) [19]. These results suggest that the redox state is both the cause and the result of the interplay between autophagy and mitochondrial turnover. Usually, antioxidant supplement is used to treat some diseases by improving redox state. N-Acetylcysteine (NAC) is a precursor to cysteine, which participates in the general antioxidant activities of the body. NAC can improve redox state and protect mitochondrial function, so as to prevent and reverse the progression of some diseases, including neurodegenerative diseases and diabetes [20–22]. However, previous work has shown that a proper mitochondrial turnover is ROS-dependent. Antioxidant supplement inhibited mitochondrial biogenesis in skeletal muscle by reducing ROS level [23, 24].

In regard to the redox regulation in skeletal muscle, previous work has shown that NAC is an effective antioxidant for treating muscular dystrophy (DMD), a severe degenerative muscle disease [25]. NAC prevents apoptosis and promotes cell survival by activating ERK pathway, so as to treat certain degenerative diseases [26]. In addition, NAC has been used as a tool for investigating the role of ROS in numerous biological and pathological processes [22]. In this study, we used antioxidant NAC to change the redox state in mice and observed their autophagic responses to short-term fasting and the basal level of autophagy in skeletal muscle. To investigate the role of antioxidant administration in autophagy, we tested the hypothesis that (1) the importance of ROS in constitutive autophagy in skeletal muscle is different from that in the stress state of fasting and that (2) mitochondrial antioxidant capacity is attenuated by NAC and involved in autophagy regulation. Our results support our hypothesis, as NAC did repress numerous markers of autophagy and fail to prevent fasting-induced autophagy in skeletal muscle. These novel findings reveal that ROS is not required for skeletal muscle autophagy resulting from fasting but important for skeletal muscle autophagy in unstressed state.

## 2. Results

### 2.1. NAC Decreases Mitochondrial ROS Production but Fails to Protect against Fasting-Induced Mitochondrial Lipid Peroxidation

**Figure 1:**
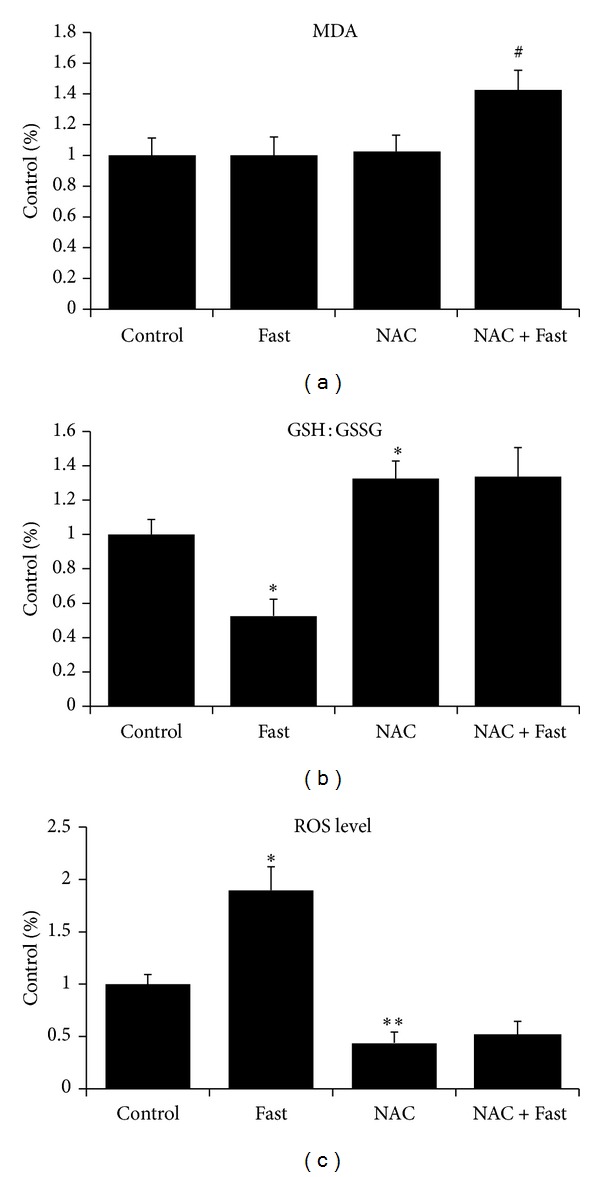
Oxidative stress biomarkers in the isolated mitochondria of gastrocnemius muscle were measured after short-term fasting. (a) MDA: methane dicarboxylic aldehyde. (b) GSH: glutathione. (c) ROS production. Values are means ± SE (fold differences). *P < 0.05, **P < 0.01 significantly different versus Control, ^#^ *P* < 0.05 significantly different versus NAC.

In our study, mitochondrial ROS production was decreased in the NAC group (*P* < 0.01) and increased in the Fast group (*P* < 0.05), compared with the Control group (Figure 1(c)). Mitochondrial GSH : GSSG was elevated in the NAC group (*P* < 0.05) and decreased in the Fast group (*P* < 0.05), compared with the Control group (Figure 1(b)). Importantly, our results show that NAC failed to protect mitochondria against fasting-induced lipid peroxidation in the NAC + Fast group (*P* < 0.05). However, in the mice without NAC treatment, fasting did not increase mitochondrial MDA level (Figure 1(a)).

### 2.2. NAC Increases Glycogen Content in Skeletal Muscle but Fails to Protect against Fasting-Induced Glycogen Consumption

**Figure 2:**
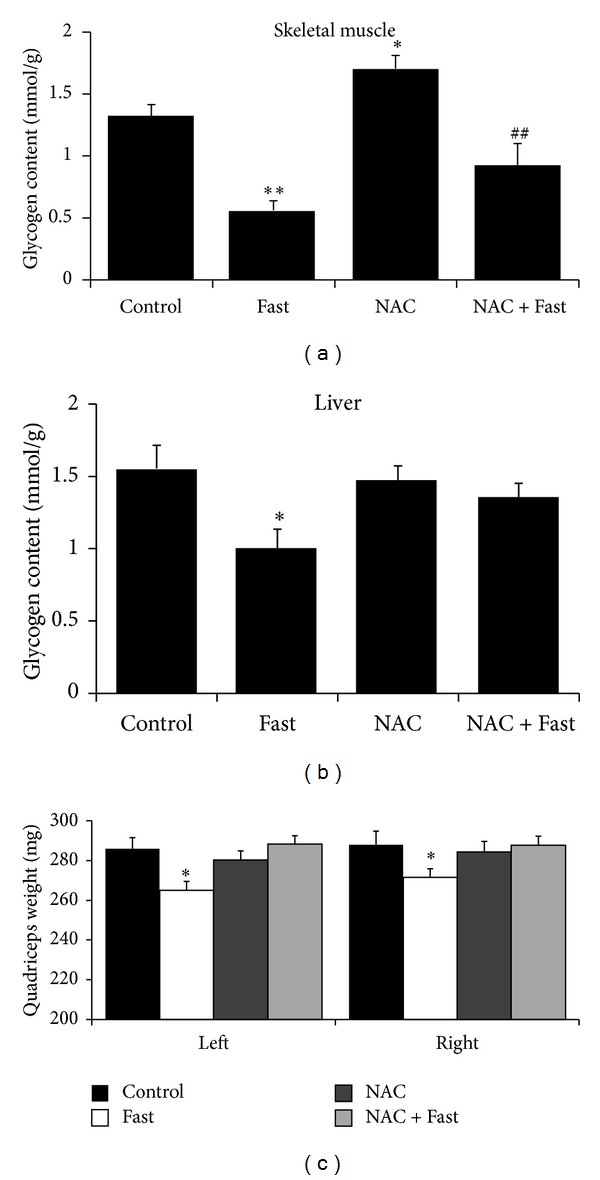
Glycogen content and quadriceps muscle weight were measured after short-term fasting. (a) Glycogen content in quadriceps muscle. (b) Glycogen content in liver. (c) Quadriceps muscle weight. Values are means ± SE. *P < 0.05, **P < 0.01 significantly different versus Control,  ^##^ *P* < 0.01 significantly different versus NAC.

Our results show that fasting reduced glycogen content in skeletal muscle, independent of NAC (*P* < 0.01, Figure 2(a)). In addition, there was a significant increase (*P* < 0.05) in skeletal muscle glycogen in the NAC group compared with Control (Figure 2(a)). Also, the glycogen content in the liver was significantly decreased (*P* < 0.05) in the Fast group compared with Control, but NAC treatment before fasting prevented this decrease (Figure 2(b)). Similarly, quadriceps muscle weight was significantly decreased (*P* < 0.05) after fasting, but NAC treatment prevented this decrease (Figure 2(c)).

### 2.3. NAC Downregulates Autophagy Markers in Skeletal Muscle but Fails to Protect against Fasting-Induced Upregulation

**Figure 3:**
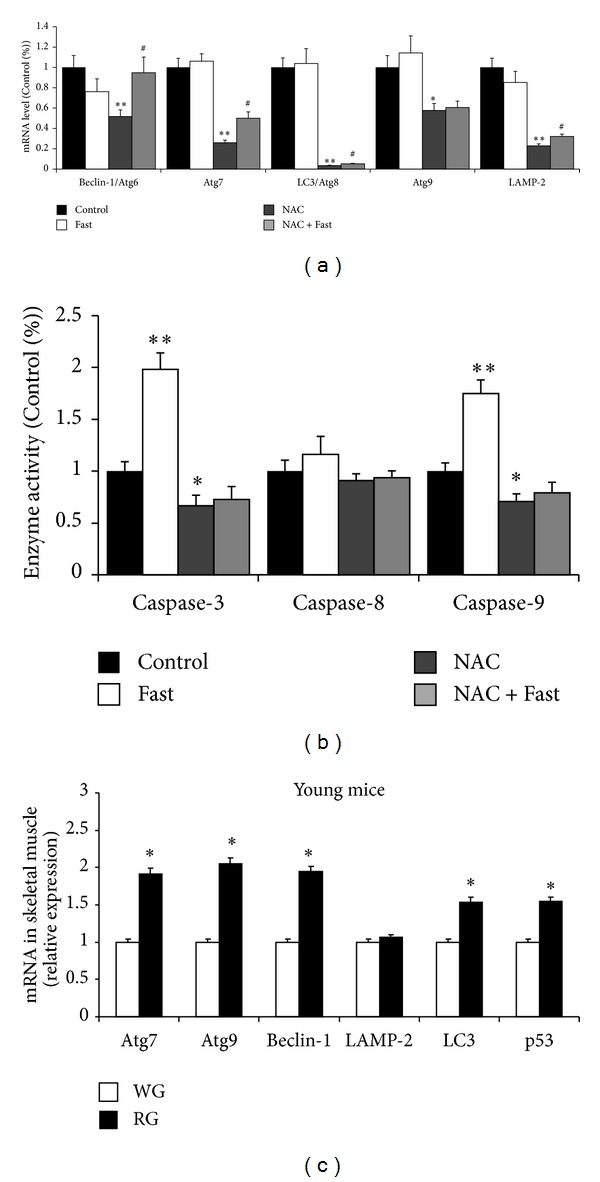
Autophagy-related gene expressions were analyzed by real-time PCR in gastrocnemius muscle. (a) mRNA level of Beclin-1, Atg7, LC3, Atg9, and LAMP2. (b) Caspases 3, 8, and 9 enzyme activity in gastrocnemius muscle. (c) Autophagy-related gene expressions in red gastrocnemius (RG) versus white gastrocnemius (WG). Values are means ± SE (fold differences). *P < 0.05, **P < 0.01 significantly different versus Control, ^#^ *P* < 0.05 significantly different versus NAC.

Our results show that mRNA expression of these Atgs in skeletal muscle was significantly decreased in the NAC group compared with Control. However, mRNA expression of those genes except Atg9 was increased significantly in the NAC + Fast group compared with NAC group (Figure 3(a)). We observed that the activity of caspases 3 and 9 was significantly decreased in the NAC group compared with Control, and fasting significantly increased the activity of caspases 3 and 9 in vehicle-treated mice (Figure 3(b)). Approximately, red gastrocnemius muscle had a higher mRNA expression of Atgs than white gastrocnemius muscle (Figure 3(c)).

### 2.4. NAC Represses TIGAR Expression but Fails to Protect against Fasting-Induced LC3 Lipidation

**Figure 4:**
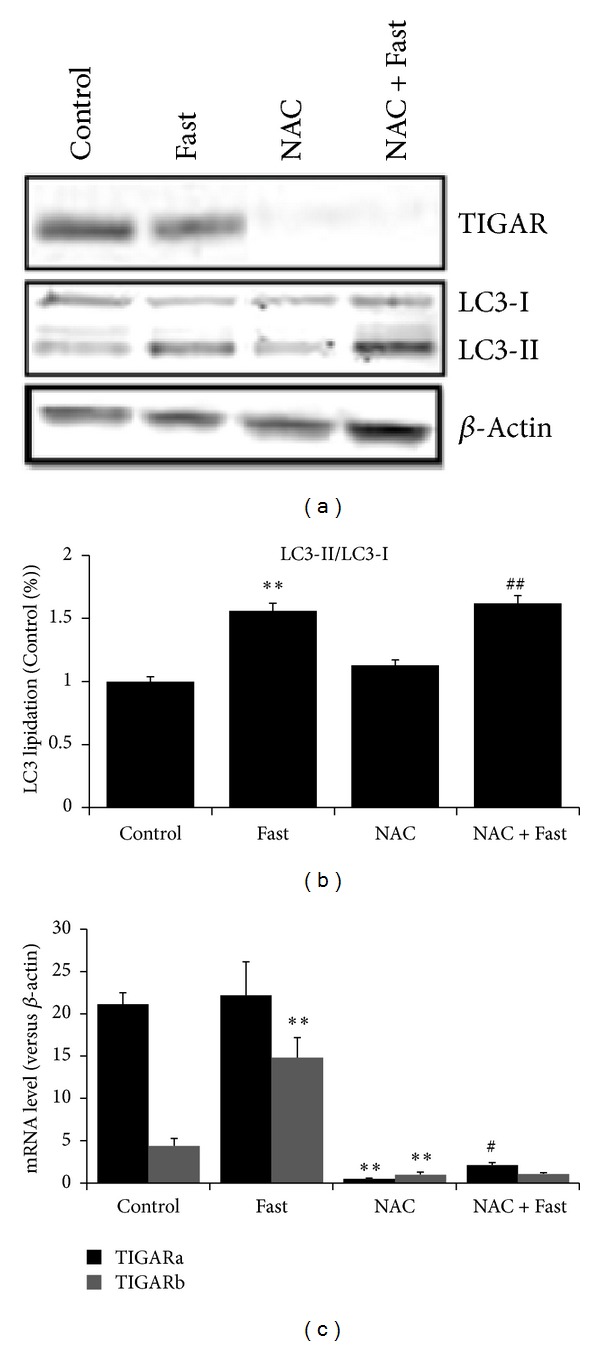
LC3 lipidation and expressions of TIGAR were measured in skeletal muscle after short-term fasting. (a) Representative western blots of LC3, TIGAR. (b) LC3-II/LC3-I ratio. (c) mRNA expression of TIGAR. Values are means ± SE. **P < 0.01 significantly different versus Control, ^#^ *P* < 0.05, ^##^ *P* < 0.01 significantly different versus NAC.

In our study, TIGAR expression was repressed significantly in the NAC group compared with Control group (*P* < 0.01, Figures 4(a) and 4(c)). TIGARb expression was significantly increased in the Fast group compared with Control group (*P* < 0.01, Figure 4(c)) and TIGARa expression was significantly increased in the NAC + Fast group compared with NAC group (*P* < 0.05, Figure 4(c)). In addition, the ratio of LC3 II to LC3 I (LC3 II/LC3 I) was also quantified as a marker of LC3 cleavage. Fasting significantly increased LC3 lipidation, independent of NAC treatment (*P* < 0.01, Figures 4(a) and 4(b)).

### 2.5. NAC Decreases MnSOD Expression and Mitochondrial Antioxidant Capacity

**Figure 5:**
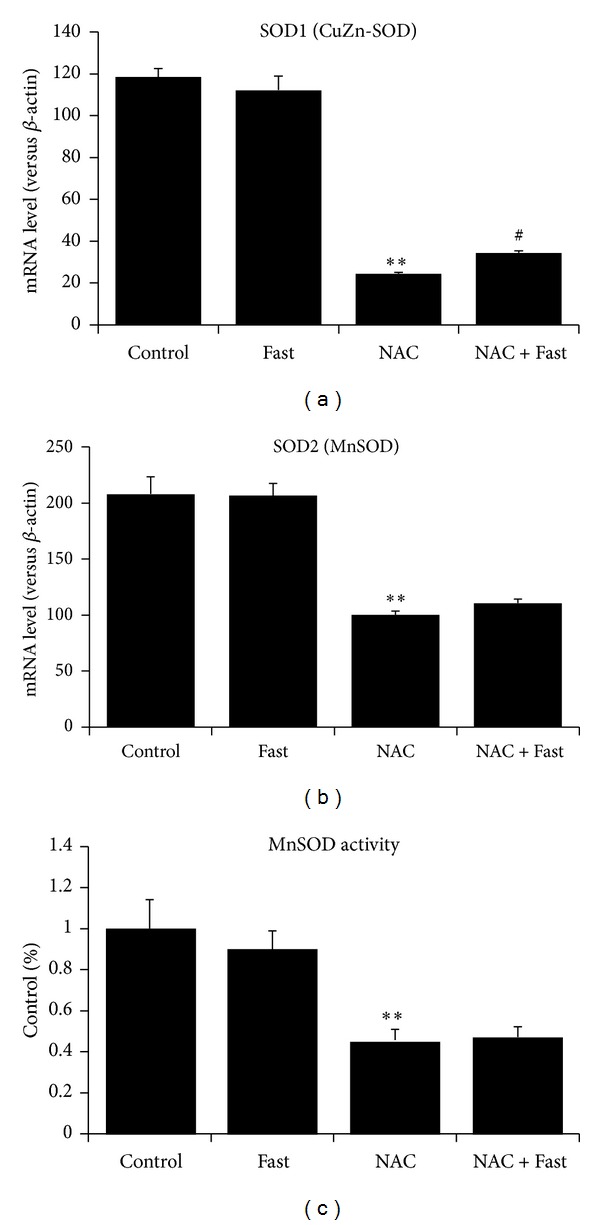
Antioxidant capacity was measured in gastrocnemius muscle after short-term fasting. (a) mRNA expression of SOD1 (CuZn-SOD). (b) mRNA expression of SOD2 (MnSOD). (c) MnSOD activity in the isolated mitochondria of gastrocnemius muscle. Values are means ± SE. *P < 0.05, **P < 0.01 significantly different versus Control, ^#^ *P* < 0.05 significantly different versus NAC.

Our results show that mRNA expression of CuZn-SOD, MnSOD in skeletal muscle was significantly decreased in the NAC group compared with Control (*P* < 0.01, Figures 5(a) and 5(b)). Similarly, MnSOD activity was significantly decreased in the NAC group compared with Control (*P* < 0.01, Figure 5(c)). Also, fasting had no effects on MnSOD expression and activity (Figure 5(c)).

## 3. Discussion

These experiments provide novel and important information regarding the effects of NAC administration on skeletal muscle autophagy. First, our findings support the hypothesis that NAC treatment dramatically decreases the expression of numerous markers of skeletal muscle autophagy at the basal level, rather than under the stress state of fasting. Further, our results reveal that NAC treatment is potential to lower mitochondrial antioxidant capacity through the inhibition of the antioxidant enzymes in mitochondria.

### 3.1. NAC Administration Depresses Skeletal Muscle Constitutive Autophagy

Significantly, constitutive autophagy at the basal level differs from stress-induced autophagy under conditions of nutrition deprivation. In stress state, enhanced autophagy favors cell survival by the degradation of cellular constituents and the recycling of macromolecules to support cellular metabolism. Glycogen in skeletal muscle and liver can be utilized to produce energy or glucose during fasting, so glycogen catabolism is also regarded as a peculiar autophagy outside lysosome [27]. In response to starvation, the activation of autophagy is essential for the most efficient degradation of glycogen [28]. In unstressed state, the basal level of autophagy in most tissues ensures the turnover of long-lived proteins and the elimination of dysfunctional organelles. Mammalian target of rapamycin complex I (mTORC1) is regarded as the dominant regulator of autophagy induction in skeletal muscle. In young TSC1- (tuberous sclerosis complex 1-) deficient mice, constitutive and starvation-induced autophagy was blocked at the induction steps via mTORC1-mediated inhibition of ULK1 (Unc-51-like kinase 1) [29]. Recent studies suggested that endogenous ROS promoted skeletal muscle autophagy at the basal level and in response to acute nutrient starvation. This effect was mediated through regulation of autophagosome initiation and PI3K/AKT inhibition [30]. Using a potential autophagy-targeting nutrient, excessive muscle autophagy in type 2 diabetic rats was ameliorated through the downregulation of ROS signaling, leading to improvement of glucose metabolism, reduction of oxidative stress, and inhibition of mitochondrial loss and dysfunction [31]. ROS scavenger NAC was reported to diminish both endoplasmic reticulum (ER) stress and autophagy markers in skeletal muscle cells [32]. Further, NAC attenuated AMPK activation under starvation condition and enhanced mTOR signaling in cervical cancer cell. The authors concluded that mitochondria-generated ROS induces autophagy mediated by the AMPK pathway under starvation conditions [33]. Consistent with the previous studies and our hypothesis, our results show that NAC significantly decreases mRNA expression of numerous markers of skeletal muscle autophagy and increases skeletal muscle glycogen. These results indicate that the basal level of skeletal muscle autophagy is ROS-dependent. Constitutive autophagy in skeletal muscle is determined by redox state. However, our results also show that NAC fails to prevent fasting-induced autophagy and glycogen reduction. Specifically, short-term fasting after NAC administration still increases the mRNA expression of numerous markers of autophagy and the level of LC3 lipidation. These results indicate that ROS-independent autophagy occurs in skeletal muscle under starvation conditions. In addition, the activation of apoptosis also contributed to muscle loss relevant to oxidative stress. Our data show that NAC blocks fasting-induced activation of caspases 3 and 9 and quadriceps muscle loss. Collectively, our results indicate that ROS is not required for fasting-induced autophagy but important for constitutive autophagy in skeletal muscle.

Additionally, our results show that the expression of most Atgs is higher in red muscle than in white muscle. This difference in constitutive autophagy may result from differential PGC-1*α* signaling and mitochondrial content in slow- and fast-twitch skeletal muscle. Two similar works at least partly support this postulation: fiber type conversion from fast to slow fibers by PGC-1*α* activates lysosomal and autophagosomal biogenesis [34], as well as mitochondrial biogenesis in skeletal muscle. PGC-1*α* overexpression increases the expression of genes related to muscle repair and autophagy [35]. Thus, we suggest that the level of constitutive autophagy in slow muscle fibers is higher than in fast muscle fibers. However, PGC-1*α* overexpression during aging protects from sarcopenia and metabolic disease by reducing apoptosis and autophagy [36]. Further studies need to determine the relationship between mitochondrial biogenesis and constitutive autophagy.

### 3.2. NAC Administration Lowers Mitochondrial Antioxidant Capacity

In regard to the effects of antioxidant supplement on skeletal muscle autophagy, we cannot ignore the role of mitochondria. On one hand, endogenous ROS is mainly generated by mitochondria; on the other hand, mitochondrial antioxidant enzyme is able to scavenge ROS and reduce oxidative stress. Recent works have suggested that mitochondrial-derived ROS induces autophagy [30, 33]; thus, mitochondrial antioxidant capacity may be responsible for the activation of autophagy. In the previous studies, NAC has been considered as a mitochondrial enhancer for improving redox status and mitochondrial dysfunction and reversing the progression of some diseases [37, 38]. Consistent with the previous results, our data show that NAC reduces mitochondrial ROS level and increases the ratio of GSH : GSSG, suggesting that reduced ROS production and elevated GSH : GSSG ratio in mitochondria are involved in the inhibition of skeletal muscle constitutive autophagy. Further, NAC in part prevents fasting-induced oxidative stress in mitochondria, but not autophagic responses, indicating that skeletal muscle autophagy under starvation is independent of mitochondrial-derived ROS. In contrast to our results in fasted mice, lower nutrient supply or calorie restriction leads to a lower production of mitochondrial ROS in skeletal muscle [39], because the lower mitochondrial membrane potential favors reducing mitochondrial ROS production [40]. In skeletal muscle of both rodents and humans, high-fat diet increases H_2_O_2_ emission of mitochondria [41]. However, in response to acute nutrient deprivation, mitochondrial ROS levels significantly increase in skeletal muscle cells [30]. These findings strongly indicate that ROS production in mitochondria is nutrient-sensitizing [42]. It is noteworthy that chronic mild nutrient restriction differs from acute nutrient deprivation in the regulation of mitochondrial ROS production. Thus, the differences in constitutive and acute stress-induced autophagy may result from differential ROS levels in skeletal muscle.

Manganese superoxide dismutase (MnSOD) is a vital antioxidant enzyme in the matrix of mitochondria by scavenging ROS. MnSOD knockdown led to chronic oxidative stress, increased autophagy, and mitochondrial biogenesis within the distal nephrons [43]. In brain, MnSOD knockdown increased oxidative stress and instead suppressed the levels of autophagy stimulators [44]. To our surprise, our results show that NAC decreases MnSOD mRNA expression and its enzyme activity. CuZn-SOD expression is also repressed. Fasting fails to rescue the decline in MnSOD expression. Our data suggest that although NAC administration reduces ROS level and increases GSH : GSSG, exogenous antioxidant supplement may lower inherent antioxidant capacity in mitochondria. ROS can lead to MnSOD upregulation through ERK2 translocation into the nucleus and dissociation of p53 from its inhibitory protein mouse double minute 2 (MDM2) [45]. Similarly, heat stress produced ROS in fibroblasts. NAC blocked the increase in MnSOD levels by heat stress through reducing ROS production [46]. Therefore, a proper level of ROS is required for MnSOD expression. Likewise, we observe that TIGAR is repressed in mRNA and protein level by NAC. Although TIGAR is not an antioxidant enzyme that eliminates ROS directly, TIGAR plays an important antioxidant role in the regulation of autophagy [47]. TIGAR inhibits glycolysis and promotes respiration. In TIGAR-expressing cells, ROS level was reduced whereas glutathione was elevated [48]. Further, TIGAR inhibited autophagy by suppressing ROS level [47]. TIGAR knockout increased mitophagy and ROS production in myocardium, followed by Bnip3 activation which is an initiator of mitophagy. Further, NAC reversed the activation of Bnip3 and mitophagy [49]. Under hypoxia, mitochondrial localization of TIGAR can control mitochondrial ROS levels and protect from cell death [50]. Therefore, TIGAR is a potential antioxidant gene for mitochondria. Collectively, our results indicate that NAC treatment with young mice attenuates the inherent antioxidant capacity in skeletal muscle, suggesting that ROS is also required for the maintenance of mitochondrial antioxidant capacity.

In summary, our study provides the first evidence that NAC decreases skeletal muscle constitutive autophagy but does not protect skeletal muscle from fasting-induced autophagy in young mice. In addition, we also showed that NAC attenuates the inherent antioxidant capacity in skeletal muscle by depressing the expression of MnSOD and TIGAR. We interpret these findings as an indication that ROS is required for the maintenance of both constitutive autophagy and antioxidant capacity in skeletal muscle. Although NAC supplement is regarded as an effective intervention for improving mitochondrial function, it should be reconsidered carefully for young and healthy individuals when NAC or the other antioxidants are used to promote muscle growth.

## 4. Materials and Methods

### 4.1. Animals

Male ICR/CD-1 mice were purchased from Sino-British Sippr/BK Lab Animal Ltd., CO (Shanghai, China) at four to five weeks of age (Young, 22.8 ± 1.2 g). Animals were housed in a temperature-controlled environment (23 ± 2°C) with 12 : 12 h light-dark cycles, where they were provided diets and water ad libitum. Mice were randomly assigned to one of four experimental groups: (1) Control, no drug treatment (Control, *n* = 8); (2) Fast, no drug treatment (Fast, *n* = 8); (3) Control, treated with NAC (NAC, *n* = 10); and (4) Fast, treated with NAC (NAC + Fast, *n* = 10). All experiments were performed in accordance with the guidelines for the use of laboratory animals published by China Ministry of Health (no. 55 order, ordained on 25 Jan., 1998). All experimental procedures were approved by the Experimental Animal Care and Use Committee at East China Normal University (ECNU 2006-05).

### 4.2. Drugs and Short-Term Fasting

The antioxidant N-acetylcysteine (NAC, Sigma) was dissolved in DMSO. The mice assigned to NAC groups received intraperitoneal injection of NAC for 3 weeks (100 mg/kg body weight/2 day). As a control, the vehicle (10% DMSO in saline) was injected to the control mice. For studies on the effects of food deprivation, the mice assigned to Fast groups were deprived of food for 24 hr before sacrifice. These mice had free access to drinking water.

### 4.3. Mitochondrial Isolation

At the end of the experimental period, animals in each group were acutely anesthetized with pentobarbital sodium. Quadriceps, gastrocnemius muscle, and liver were removed completely and weighed. For mitochondrial isolation, fresh gastrocnemius muscle was rinsed with PBS and put into ice-cold isolation buffer (0.075 M sucrose, 0.225 M sorbitol, 1 mM EGTA, 0.1% fatty acid-free bovine serum albumin (BSA), and 10 mM Tris-HCl, pH 7.4). Tissues were sheared carefully to mince, rinsed to get rid of residual blood, and then homogenized in 1 mL isolation buffer per 100 mg tissue. The homogenate was centrifuged at 1000 g for 5 min at 4°C using a Beckman centrifuge (Avanti J-26XP); the resulting supernatant was decanted and saved. The pellet was washed once with isolation buffer. The supernatant was combined and centrifuged at 9,000 g for 10 min at 4°C. The mitochondrial pellet was washed and centrifuged twice at 15,000 g for 2 min at 4°C with isolation buffer [51]. Mitochondrial protein content was assayed using BSA as a standard according to Bradford.

### 4.4. Mitochondrial ROS Generation and Antioxidant Capacity

The dichlorofluorescin diacetate (DCF-DA) was used as a probe to detect mitochondrial ROS generation. The assay was performed on freshly isolated mitochondria. Samples (30 *μ*g proteins) were incubated with 10 *μ*L of DCF-DA (10 *μ*mol/I) for 3 h at 37°C. The fluorescent product formed was quantified by spectrofluorometer at the 485/525 nm. Changes in fluorescence were expressed as arbitrary unit. To assay mitochondrial lipid peroxidation, MDA levels were measured using an assay kit according to the manufacturer's protocol (Nanjing Jiancheng Biotech, Nanjing, China). Briefly, isolated mitochondria were incubated at 37°C for 3 h, treated with buffer, and centrifuged at 10000 ×g for 15 min. The supernatants were incubated with thiobarbituric acid (TBA), and the absorbance of the supernatants was measured using a spectrophotometer at a wavelength of 535 nm. Also, the GSH/GSSG ratio and MnSOD activity were measured using an assay kit (Nanjing Jiancheng Biotech, Nanjing, China) according to the manufacturer's protocol, respectively.

### 4.5. Caspases Activity

Caspases 3, 8, and 9 activity was determined with an assay kit (Beyotime, China) according to the manufacturer's instructions. Under the caspase-3, acetyl-Asp-Glu-Val-Asp p-nitroanilide (Ac-DEVD-pNA) can be changed into the yellow product, p-nitroaniline (pNA). Under the caspase-8, acetyl-Ile-Glu-Thr-Asp p-nitroanilide (Ac-IETD-pNA) can be changed into the yellow product, pNA. Under the caspase-9, acetyl-Leu-Glu-His-Asp p-nitroanilide (Ac-LEHD-pNA) can be changed into the yellow product, pNA. Finally, pNA content was measured using the spectrophotometer at an absorbance of 405 nm. Protein content was assayed using BSA as a standard according to Bradford.

### 4.6. Glycogen Content

Glycogen content was determined with an assay kit (Nanjing Jianchen Biotech, China) according to the manufacturer's instructions.

### 4.7. Real-Time PCR

Total RNA was prepared from ~100 mg of frozen tissues using TRIzol (Invitrogen, Chromos, Singapore) and purified according to the instructions included. Double-stranded cDNA was synthesized from ~1 *μ*g of total RNA using ReverTra Ace qPCR RT kit (TOYOBO, Osaka, Japan). Real-time PCR reactions were set up using the SYBR-Green PCR kit (TOYOBO, Osaka, Japan) and were cycled in StepOne Real-Time PCR System (Applied Biosystems, CA, USA) as previously described [52]. The abundance of target mRNA was normalized to that of *β*-actin. Primer pairs were designed based on GenBank reference sequences and listed in Table S1 available online in Supplementary Material at http://dx.doi.org/10.1155/2014/315896.

### 4.8. Western Blotting

The isolated muscle tissue (~200 mg) was weighed and cut into pieces at 4°C, and 2 mL (10× dry weight of isolated muscle fibers) of ice-cold buffer (50 mM Tris HCl, 150 mM NaCl, 1 mM EDTA, 0.2 mM PMSF, 1% NP-40, pH 7.4, cocktail) was added. The tissue pieces were then homogenized, after which homogenates were centrifuged for 10 min at 8,000 g and 4°C. The total protein content of the supernatant was quantified using bicinchoninic acid reagents and BSA standards (Shanghai Sangon, Shanghai, China). The protein samples were immunoblotted as previously described [52]. Visualization of reaction bands was performed by 3,3′-diaminobenzidine staining (Shanghai Sangon, China) and scanned densitometrically. *β*-Actin was used to standardize for the amount of protein loaded.

### 4.9. Statistical Analysis

Data are reported as mean ± SEM. Statistical differences between treatments were determined using ANOVA. For all tests the significance level was set at *P* < 0.05.

## Supplementary Material

Primers and antibodies. The primers used for PCR were designed based on GenBank reference sequences and listed as follows, and the primary antibodies used for Western blot were obtained from Santa Cruz.
