# Supplementary material for: Antioxidant Supplement Inhibits Skeletal Muscle Constitutive Autophagy rather than Fasting-Induced Autophagy in Mice
Source: Oxid Med Cell Longev. 2014 Jun 15;2014:315896. doi: 10.1155/2014/315896 (PMC4084590; doi:10.1155/2014/315896)
Supplement: Supplementary file 1 — Primers and antibodies. The primers used for PCR were designed based on GenBank reference sequences and listed as follows, and the primary antibodies used for Western blot were obtained from Santa Cruz. [file 315896.f1.doc]

1. **PCR Primers**

| **Genes** | **Primer Sequence** |
| --- | --- |
| Atg7 Forward | ttgtagcacctgctgacctg |
| Atg7 Reverse | tgcaggacagagaccatcag |
| Atg9 Forward | gtgcttattgccctcaccat |
| Atg9 Reverse | ggcatgtagtggatgtgtgc |
| Beclin1 Forward | ggccaataagatgggtctga |
| Beclin1 Reverse | gctgcacacagtccagaaaa |
| LAMP-2 Forward | cacccactccaactccaact |
| LAMP-2 Reverse | ttgtggcagggttgatgtta |
| LC3 Forward | catgagcgagttggtcaaga |
| LC3 Reverse | ttgactcagaagccgaaggt |
| p53 Forward | gtaggaaggcgcgtggtag |
| p53 Reverse | cagttacaggaaccccgag |
| SOD1 Forward | agatgacttgggcaaaggtg |
| SOD1 Reverse | tctccatcagctgtcattgc |
| SOD2 Forward | gccccctgagttgttgaata |
| SOD2 Reverse | agacaggcaaggctctacca |
| TIGAR Forward | cctaatgagttgaatgacag |
| TIGAR Reverse | attggaaattgtggaggtg |
| β-actin Forward | tgttaccaactgggacgaca |
| β-actin Reverse | ctatgggagaacggcagaag |

1. **Antibody**

| Target | No. | Company |
| --- | --- | --- |
| LC3-II | sc-16755 | Santa Cruz |
| TIGAR | **sc-68239** | Santa Cruz |
| β-actin | **sc-81178** | Santa Cruz |
